# Standardized image evaluation in patients with idiopathic normal pressure hydrocephalus: consistency and reproducibility

**DOI:** 10.1007/s00234-019-02273-2

**Published:** 2019-08-10

**Authors:** Karin Kockum, Johan Virhammar, Katrine Riklund, Lars Söderström, Elna-Marie Larsson, Katarina Laurell

**Affiliations:** 1 Department of Pharmacology and Clinical Neuroscience, Neurology, Östersund, Umeå University, SE-901 87 Umeå, Sweden; 2 Department of Neuroscience, Neurology, Uppsala University Hospital, SE-751 85 Uppsala, Sweden; 3 Department of Radiation Sciences, Diagnostic Radiology, Umeå University, SE-901 87 Umeå, Sweden; 4 Unit of Research, Education and Development, Östersund Hospital, SE-831 31 Östersund, Sweden; 5 Department of Surgical Sciences, Radiology, Uppsala University, SE-751 85 Uppsala, Sweden

**Keywords:** Hydrocephalus, normal pressure, Magnetic resonance imaging, Tomography, x-ray computed, Observer variation

## Abstract

**Purpose:**

Assess the agreement for two investigators between computed tomography (CT) and magnetic resonance imaging (MRI) for seven imaging features included in the iNPH Radscale, a radiological screening tool.

**Methods:**

The study included 35 patients with idiopathic normal pressure hydrocephalus (iNPH) who were treated surgically from 2011 to 2015 at Uppsala University Hospital with preoperative CT and MRI performed with maximum 3 months between scans. Seven features were assessed: Evans’ index, temporal horn size, callosal angle, periventricular white matter changes, narrow high convexity sulci, focally enlarged sulci, and enlarged Sylvian fissures. All scans were assessed by two investigators who were blinded to each other’s results and to clinical data.

**Results:**

The agreement between CT and MRI was almost perfect for Evans’ index, temporal horns, narrow sulci, and Sylvian fissures (kappa and intraclass correlation, 0.84–0.91, *p* ≤ 0.001). There was substantial to almost perfect agreement for callosal angle and focally enlarged sulci. The concordance between modalities was fair for changes in periventricular white matter.

**Conclusion:**

CT and MRI are equally good for assessing radiological signs associated with iNPH except for periventricular white matter changes, as MRI has superior soft tissue contrast. The other imaging features can be evaluated consistently, and assessments are reproducible independent of modality. Therefore, the iNPH Radscale is applicable to both CT and MRI and may become an important tool for standardized evaluation in the workup in patients with suspected iNPH.

**Electronic supplementary material:**

The online version of this article (10.1007/s00234-019-02273-2) contains supplementary material, which is available to authorized users.

## Introduction

Idiopathic normal pressure hydrocephalus (iNPH) is a progressive neurological syndrome that is characterized by impairments in gait, balance, cognition, and urinary bladder control [1]. The diagnosis of iNPH is based on the presence of symptoms as well as on ventriculomegaly and related imaging findings [2, 3]. The etiology is not clear, but the cerebrospinal fluid dynamics are disturbed, leading to the accumulation of cerebrospinal fluid in the brain [4].

There are several imaging features that are typical of iNPH, such as widening of the temporal horns, small callosal angle, periventricular white matter changes, narrowing of the parafalcine sulci over the vertex, focally enlarged sulci, and widening of the Sylvian fissures [5–11]. The iNPH Radscale, is a radiological scale that summarizes these imaging features into a structured score. We have previously showed that the iNPH Radscale score correlates with iNPH symptoms in an unselected population [12].

According to the two guidelines, computed tomography (CT) can be used in the diagnostics of iNPH, but magnetic resonance imaging (MRI) is preferred due to its superior soft tissue contrast, lack of ionizing radiation [2, 3], and possibility to discriminate between iNPH and obstructive hydrocephalus [13]. However, patients with iNPH symptoms commonly undergo a CT brain first, due to fall accidents or through referral from physicians in the general health care. Since the condition can be effectively treated by shunt surgery, especially in the early stages of the disease, it is important that the radiological picture of iNPH is recognized on CT as well as on MRI [14, 15].

The inter-rater agreement for iNPH-associated radiological features in one modality has been described (see Table 1) [16, 17, 19–21], but none have directly compared CT and MRI for evaluating patients with iNPH. The aim of this study was to directly compare CT and MRI for assessing the seven imaging features of iNPH mentioned above by determining the inter- and intra-rater agreement. We also compared the reliability of each imaging feature alone to that of the overall iNPH Radscale score.

**Table 1:** Inter-rater agreement for iNPH-associated radiological features as reported by previous studies

Radiological feature	Modality	Study	Concordance	Readers *n*	Patients *n*
Evans’ index					
	CT	Bao [13]	0.874 (ICC)	2	36 (NPH)
	MRI	Miskin [14]	0.82 (ICC)	3	106 (36 NPH, 34 AD, 36 C)
	MRI	Reinard [15]	0.913 (ICC)	5	30 (NPH)
Callosal angle					
	CT	Bao [13]	0.570 (ICC)	2	36 (NPH)
	MRI	Miskin [14]	0.91 (ICC)	3	106 (36 NPH, 34 AD, 36 C)
	MRI	Reinard [15]	0.865 (ICC)	5	30 (NPH)
Temporal horns					
	CT	Bao [13]	0.469 (ICC)	2	36 (NPH)
	MRI	Reinard [15]	0.729 (ICC)	5	30 (NPH)
White matter changes					
3-step scale	CT	Van Swieten [16]	0.63 (Wkappa)	11	24 (NPH)
3-step scale	MRI	Van Swieten [16]	0.78 (Wkappa)	5	24 (NPH)
ARWMC	CT	Wahlund [17]	0.67 (kappa)	4	77 (NPH)
ARWMC	MRI	Wahlund [17]	0.48 (kappa)	4	77 (NPH)
DESH					
	MRI	Akigushi [18]	0.89 (?)	2	84 (NPH)

*AD* Alzheimer’s disease, *ARWMC* age-related white matter changes, *C* controls, *DESH* disproportionally enlarged sulci hydrocephalus, *ICC* intraclass correlation, *NPH* normal pressure hydrocephalus, *Wkappa* weighted kappa

## Methods

**Fig. 1:**
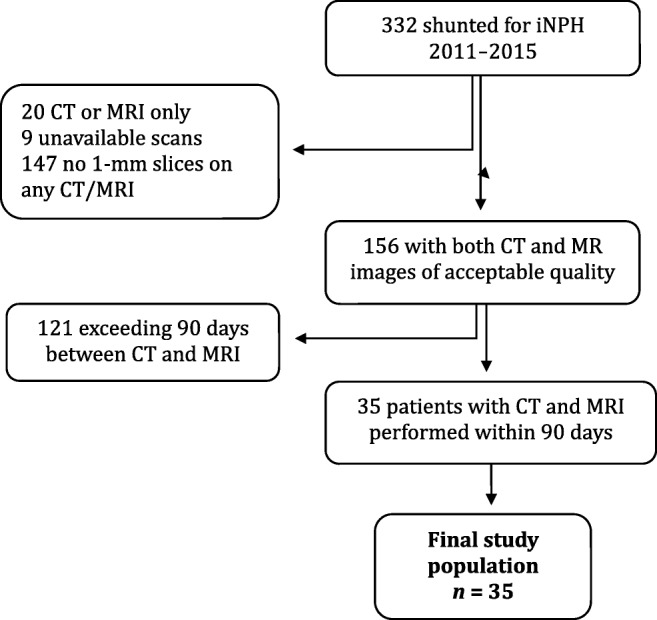
Flowchart showing patient selection

The selection process of the final study population (*n* = 35) is illustrated in Fig. 1. During 2011 to 2015, 332 patients were diagnosed with iNPH and treated by the surgical placement of a ventriculoperitoneal shunt at Uppsala University Hospital. Selection for shunt surgery was based on the evaluation of a multidisciplinary hydrocephalus team. All patients had ventriculomegaly and clinical signs of iNPH, i.e., impaired gait in combination with cognitive deficiency and/or incontinence symptoms. The diagnostic evaluation included lumbar puncture with analyses of CSF and tap test. The majority underwent infusion test as well. Biomarkers of CSF were analyzed for differential diagnoses such as Alzheimer’s disease.

The criteria for inclusion in the present study were a preoperative MRI and CT examination of the brain with maximum 3 months between scans that fulfilled the following minimal technical requirements:

- MRI: T2-FLAIR (3D with approximately 1-mm voxel size or 2D with maximum slice thickness of 5 mm). If it was a 2D sequence, we also required a 3D T1-weighted sequence with approximately 1-mm voxel size or a coronal T2-FLAIR or T1-weighted sequence with maximum slice thickness of 4 mm.
- CT: an isotropic voxel size of maximum 1 mm or a coronal 4-mm reformat was required. If thin sections were not accessible, the available 4-mm reconstructions in three planes were used.

In total, 35 patients fulfilled the above inclusion criteria. The majority of the CT and MRI scans (71%; *n* = 25) were performed in Uppsala, whereas 29% (*n* = 10) were performed at the referring hospital within the health care region.

Two investigators independently evaluated all scans: one was a fifth-year radiology resident (reader A) and one was a senior consultant in neuroradiology with 35 years of experience (reader B). They were blinded to patient pre- and postoperative clinical data but were aware that all patients received a shunt after imaging. The radiological features were scored separately for CT and MRI for each patient, and reader A re-assessed all images after a minimum of 6 weeks. Both readers assessed all images using OsiriX MD software version 9.0.2 (Pixmeo SARL). Assessments were performed using multiplanar reconstruction (MPR) mode. To standardize measurements, the planes were carefully aligned with anatomical landmarks in all three dimensions. The transverse plane was positioned parallel to the pituitary-fastigium (of the fourth ventricle) axis. The coronal plane was angulated perpendicular to the transverse plane for all measurements except for the callosal angle, which required a coronal plane perpendicular to the intercommissural plane. The sagittal plane was parallel to the falx cerebri.

Ventriculomegaly was graded using Evans’ index, dividing the maximal width of the frontal horns by the inner diameter of the skull in the same transverse section [22]. Temporal horn width was measured in the transverse plane and was reported as the mean value of the left- and right-side widths. The callosal angle was measured in the coronal plane through the posterior commissure and perpendicular to the intercommissural plane [6]. Periventricular white matter changes were reported as not present, as caps around the frontal horns, or as confluent areas, corresponding to Fazekas grade 0, 1, and 2–3 [23]. Narrow parietal sulci were reported as not present, present in the parafalcine region, or as extending along the vertex [9]. Focally dilated sulci were reported as not present or present [24]. Sylvian fissures were considered dilated when they were wider than the surrounding sulci [11]. The iNPH Radscale score was calculated (see electronic supplementary material for an imaging atlas with cutoff values for the scoring levels).

### Statistics

Descriptive statistics are presented as medians and interquartile ranges. The Mann-Whitney *U* test and *χ*^2^ test when appropriate were used to investigate differences between groups, and the significance level was set at *p* < 0.05. Inter- and intra-rater reliability was calculated using Cronbach’s alpha, interclass correlation, and kappa statistics, including weighted kappa and prevalence-adjusted bias-adjusted kappa. The kappa values were interpreted according to Landis and Koch: 0.01–0.20 slight agreement; 0.21–0.40 fair agreement; 0.41–0.6 moderate agreement; 0.61–0.80 substantial agreement; and 0.81–1.00 almost perfect agreement [25]. Bland-Altman plots illustrated the differences between measurements. Statistical analyses were performed using SPSS version 25 (IBM Corp, Armonk, NY, USA).

## Results

The final sample consisted of 35 patients with a median age of 73 years (IQR 70–78). The sex distribution was even (51% men, 49% women), with no significant difference in age (men median 73.5 years, IQR 70–78; women 73 years, IQR 70–79) and no significant differences in the intervals between CT and MRI evaluation (CT: median 74 days, IQR 71–86 for men and median 82 days, IQR 74–86 for women; MRI: median 108 days, IQR 46–116 for men and median 116 days, IQR 108–116 for women). Out of the 35 participants, 12 CT scans had 1-mm sections available, and 31 of the MRI included a 3D sequence. At 1-year follow-up, 15 patients where defined as responders and 18 as non-responders. Shunt response was defined as a patient improved in any of the following 3 criteria:

1. Twenty percent reduction in the time or the number of steps in at least one of the 2 motor function tests (Timed Up and Go Test and 10-m walk measured in steps and seconds)
2. Four levels in the Mini-Mental State Examination
3. One level in an ordinal continence scale and improvement in the Mini-Mental State Examination score of 2 levels

Two patients where lost at follow-up at Uppsala University Hospital, where one was a non-regional citizen and one only had follow-up at 3 months.

### Agreement between investigators

Table 2 shows the inter-rater agreement for CT and MRI assessment of the seven radiological features associated with iNPH. Notably, inter-rater agreement was substantial to almost perfect for assessment of Evans’ index (concordant readings for CT and MRI in 97% of the cases); for assessment of the size of the temporal horns (concordant readings for CT and MRI in 83% of the cases); for assessment of narrow sulci (concordant readings in 86% of the cases for CT and in 89% of the cases for MRI); and for assessment of enlarged Sylvian fissures (concordant readings for CT and MRI in 91% of the cases). There was almost perfect inter-rater agreement for assessment of focally enlarged sulci for CT (concordant readings in 91% of the cases) and fair inter-rater agreement for MRI (concordant readings in 74% of the cases). Inter-rater agreement was fair to substantial for assessment of the callosal angle (concordant readings in 74% of the cases for CT and in 83% of the cases for MRI). The inter-rater agreement was fair for assessment of periventricular white matter changes (concordant readings in 60% of the cases for CT and in 57% of the cases for MRI).

**Table 2:** Intra- and inter-rater agreement between consecutive assessments by CT and MRI

	CT		CT vs. MRI		MRI	
	Intra-rater agreement	Inter-rater agreement	Intra-rater agreement		Intra-rater agreement	Inter-rater agreement
	Reader A	Reader A vs. Reader B	Reader A	Reader B	Reader A	Reader A vs. Reader B
	(95% CI)	(95% CI)	(95% CI)	(95% CI)	(95% CI)	(95% CI)
Evans’ index (ICC)	0.87 (0.76–0.93)	0.81 (0.46–0.92)	0.88 (0.63–0.95)	0.84 (0.66–0.92)	0.88 (0.75–0.94)	0.75 (0.4–0.88)
Temporal horns (ICC)	0.90 (0.77–0.96)	0.78 (0.61–0.88)	0.87 (0.75–0.93)	0.85 (0.73–0.92)	0.78 (0.60–0.88)	0.73 (0.47–0.86)
Callosal angle (ICC)	0.80 (0.60–0.90)	0.54 (− 0.03–0.80)	0.74 (0.54–0.86)	0.88 (0.78–0.94)	0.81 (0.66–0.90)	0.62 (− 0.07–0.86)
WMC (Wkappa)	0.67 (0.63–0.90)	0.50 (0.23–0.76)	0.49 (0.28–0.94)	0.54 (0.35–0.74)	0.57 (0.39–0.74)	0.49 (0.24–0.74)
Narrow sulci (Wkappa)	0.67 (0.41–0.92)	0.62 (0.39–0.86)	0.91 (0.79–1.00)	0.89 (0.71–1.00)	0.74 (0.43–1.05)	0.75 (0.48–1.02)
Sylvian fissures (PABAK)	0.68 (0.39–0.97)	0.68 (− 0.18–1.55)	0.72 (− 0.08–1.51)	0.52 (− 0.72–1.77)	0.60 (− 0.35–1.54)	0.62 (− 0.37–1.62)
	0.77	0.83	0.83	0.83	0.77	0.83
Enlarged sulci (PABAK)	0.67 (0.34–1.00)	0.68 (− 0.22–1.57)	0.52 (− 0.22–1.26)	0.36 (− 0.81–1.54)	0.24 (− 0.63–1.12)	0.36 (− 0.37–1.09)
	0.83	0.83	0.60	0.71	0.49	0.49

*ICC* intraclass correlation, *PABAK* prevalence-adjusted bias-adjusted kappa, *Wkappa* weighted kappa, *WMC* white matter changes

### Agreement over time and modalities for one investigator

**Fig. 2:**
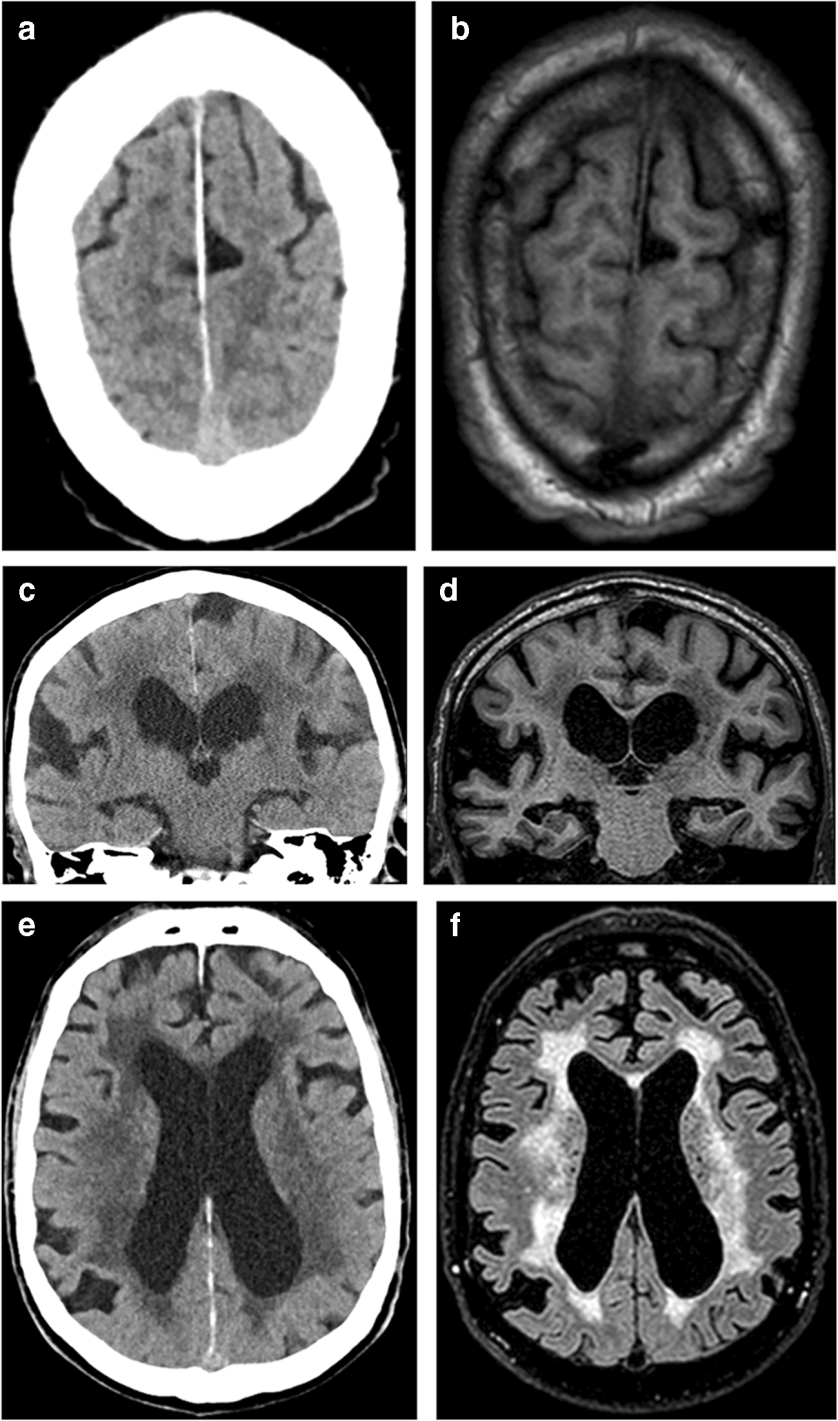
CT images and T1-weighted MR images of the same patient illustrate narrow sulci and focally enlarged sulci (**a**, **b**) and dilated Sylvian fissures (**c**, **d**). The CT image and T2-FLAIR-weighted MR image of the same patient show periventricular white matter changes (**e**, **f**)

**Fig. 3:**
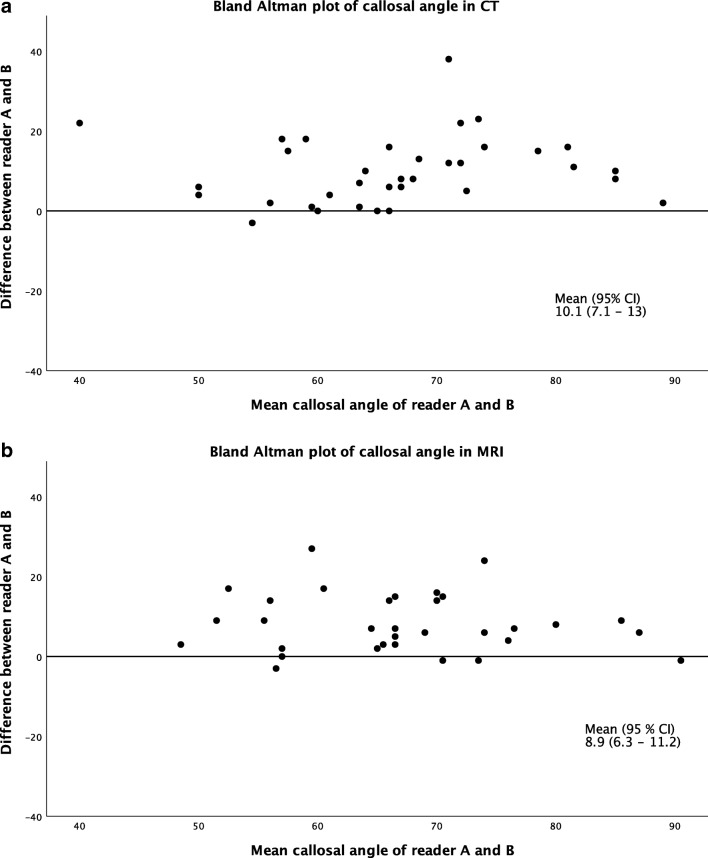
Bland-Altman plot of the callosal angle as assessed by readers A and B by CT (**a**) and MRI (**b**)

Intra-rater agreement (test-retest reliability) was equally distributed for both modalities, with higher values for continuous data (Table 2). Figure 2 shows examples of comparable CT and MRI scans in the same patient in whom the assessment remained the same, independent of the modality. When the results were compared for different modalities for the same reader, the intra-rater agreement was lower for white matter changes and for focally enlarged sulci. Callosal angle assessment showed almost perfect intra-rater agreement for both CT and MRI, and intra-rater agreement was only slightly lower when the two modalities were compared for the same reader (Table 2). However, when comparing the two readers, the inter-rater agreement was fair to moderate, and Bland-Altman plots (Fig. 3) showed a systematic difference between the readers, with reader B having a mean score that was lower by 10°.

### Agreement between readers and modalities for iNPH Radscale

When summarizing the parameters of the iNPH Radscale score, the median score was 9 (IQR 8–10) for both readers and modalities. The intra-rater reliability varied from moderate to almost perfect, and the inter-rater reliability, as assessed by weighted kappa, was substantial (Table 3). The intra-rater agreement, as assessed by Cronbach’s alpha, had high values that ranged from 0.79 to 1.0. Comparing readers, Cronbach’s alpha was marginally lower, ranging from 0.79 to 0.85 (Table 3).

**Table 3:** Intra- and inter-rater agreement for the iNPH Radscale

Intra-rater agreement	Cronbach’s alpha	Wkappa (95% CI)
Reader A		
CT: test-retest	0.85	0.74 (0.60–0.88)
MRI: test-retest	0.79	0.65 (0.49–0.80)
CT vs. MRI: internal consistency	0.82	0.56 (0.37–0.74)
Reader B		
CT vs. MRI: internal consistency	1.0	1.0 (1.0–1.0)
Inter-rater agreement		
CT reader A-CT reader B	0.85	0.74 (0.60–0.88)
MRI reader A-MRI reader B	0.79	0.62 (0.45–0.80)

*Wkappa* weighted kappa

## Discussion

In this retrospective study, the agreement between investigators, evaluations, and modalities for seven radiological features associated with iNPH was substantial to almost perfect, with the exception for periventricular white matter changes and, to some extent, focally enlarged sulci.

### Individual features

With standardized projections, the measurements can be performed in a more uniform way and with high reliability, as shown in this study for Evans’ index and temporal horns. Evans’ index is a robust measurement that showed almost perfect agreement between readers, both in this study and in previous studies (Table 1). Furthermore, the concordance between CT and MRI assessment was high, with an ICC over 0.80.

Measurements of the temporal horns showed higher inter-rater agreement in this study than in earlier publications, especially for CT [16, 19]. Thin sections, proper alignment, and a consensus on where to measure are important factors that increase agreement. Very few studies report the agreement for temporal horns as measured in millimeters, as in the present study, but more often this is reported as mild, moderate, and severe dilation. In this study, the substantial to almost perfect agreement for temporal horns was stable both between modalities and between readers.

**Fig. 4:**
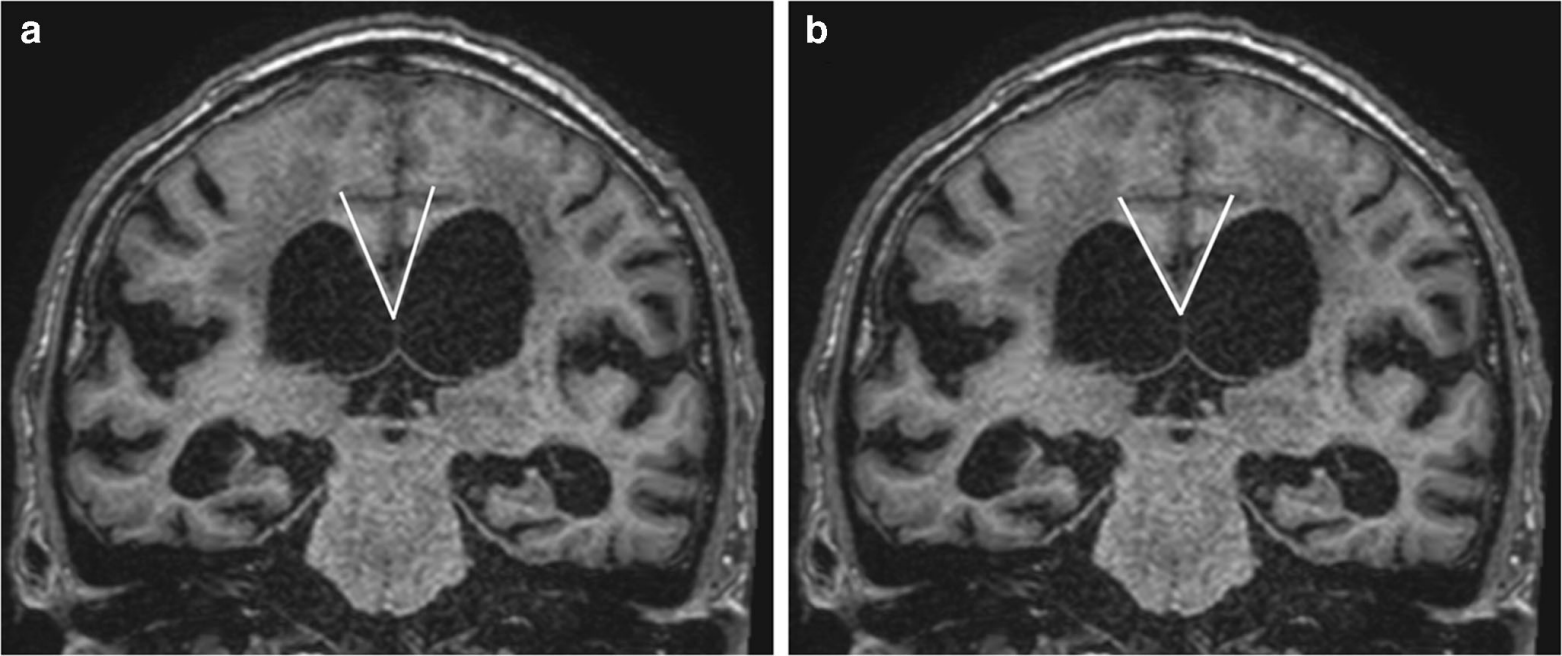
Coronal T1-weighted MR images show the callosal angle measured in the same coronal section with tangents in different positions along the curvature of the ventricular roof

The assessment of the callosal angle was robust in terms of test-retest reliability and consistency between CT and MRI. The inter-rater reliability is in line with that reported by Bao et al. [16] but lower than that reported by Miskin et al. [17] and Reinard et al. [19]. However, in Miskin et al., coronal reformatting was performed beforehand and supplied to all readers. In the present study, the two readers individually angulated the coronal reformat perpendicular to the commissure plane, which could affect agreement. The systematic 10° difference between the readers (Fig. 3) is a good example of the importance of harmonization between readers. Not only is the alignment perpendicular to the commissure plane of great importance but also is the placement of the tangent along the ventricular roof. When the callosal angle is small, the curvature of the ventricular roof is more pronounced, thereby making precise measurement of the callosal angle more difficult (Fig. 4).

The assessment of periventricular white matter changes varied to the same extent as in previous studies (Table 1). It was expected that the periventricular white matter changes would have low agreement between modalities, since MRI has a higher sensitivity for detecting white matter changes. However, the clinical relevance of this parameter is uncertain, since white matter changes should not exclude patients from shunting [26]. Furthermore, although periventricular white matter changes are a supporting feature of iNPH according to the diagnostic guidelines, this is also a common finding in elderly patients with vascular disease that is not restricted to iNPH. In the clinical setting, changes over time in the same patient are compared with the same modality of examination, i.e., MRI or CT, to limit discrepancies.

For the three sulci-related parameters, the intra- and inter-rater agreement was substantial for CT and MRI, with one exception. Reader A had lower intra-rater agreement for focally enlarged sulci on MRI than on CT as well as when CT was compared with MRI, which is interpreted as an expression of inexperience. The scale was first developed on CT, and Reader A was more used to assessing that modality. In a patient with borderline compression of sulci, the lower resolution of CT blurs the contours of the sulci, potentially overestimating the narrowing and therefore increasing the likelihood of concordance between readers. MRI defines the border between CSF and gray matter, which hypothetically could make two readers differ in their scoring. Features that involve more subjective assessments require distinct predefined rules to achieve high reliability. Narrow sulci and focally enlarged sulci should be assessed both in the most superior transverse slices and in the coronal view. Widening of the Sylvian fissures is usually evident in the coronal plane but should also be assessed in sagittal images, where the comparison with surrounding sulci is facilitated.

### INPH Radscale compared with individual features

We also compared the ratings for the seven features with the total scores on the iNPH Radscale [12]. The two investigators had the same median and range for both modalities, indicating concordant assessments. The results however demonstrated less consistent reliability for the iNPH Radscale than for the separate parameters (Table 3). The agreement was moderate to almost perfect for one reader using the same modality (test-retest reliability), whereas inter-rater agreement was moderate to substantial. This may be due in part to the fact that the iNPH Radscale has 13 levels, whereas each radiological parameter has either two or three levels. The likelihood of deciding on the exact same score will therefore decrease and thus also decrease the absolute agreement. However, the test-retest reliability and interrater agreement according to Cronbach’s alpha were still acceptable for both CT and MRI, exceeding the rule-of-thumb threshold level of 0.7 [18].

There was better agreement for the callosal angle with continuous measurements than with the iNPH Radscale score. This may be because many subjects had a callosal angle of about 60°, which is the cutoff between one or two points. A difference of just 1° between the two readers, for example, 59 vs. 60 degrees, can lead to a different score.

The underestimation of periventricular white matter changes on CT is a potential problem when comparing different modalities. According to the diagnostic guidelines, periventricular edema is a supportive feature of iNPH and does not alter the treatment plan. Thus, it may be excluded in a future revision of the iNPH Radscale, at least for CT.

### Limitations

Agreement studies have a blinded protocol, so one cannot compare modalities or present and previous readings. This contrasts with the clinical setting, in which the comparison of images is a key strategy of high-quality reporting by the radiologist. Therefore, some of the discrepancies found in this study would probably be less important in the daily clinical routine.

During the patient selection process, 297 patients were excluded because of the inclusion criteria, i.e., a short time interval between investigations and the use of thin sections or data that were reformatted in three planes. However, if there was a longer time interval between the examinations, the morphology might have changed due to disease progression, which would have affected the results. Likewise, lower image quality would have influenced the accuracy of the measurements and visual scoring.

The sample included scans suited for multiplanar reconstruction (MR 3D and CT in 1-mm sections) as well as coronal reformats saved to PACS from the scanner. The coronal reformat was then re-angulated perpendicular to the commissure plane using multiplanar reconstruction, to correctly measure the callosal angle. The thin sections are of course preferable for correct alignment, but the coronal reformat realigned is still an improvement. The exclusion would have been too big if 3D sequence for MR and 1-mm sections for CT had been mandatory.

Due to the selected sample population, the prevalence of radiological changes was high. Indeed, a more diverse sample with a corresponding number of positive and negative cases would have simplified the calculations. Skewed samples, which are common in clinical materials, complicate the interpretation of kappa and therefore require compensation in the statistical method. Since the kappa values were possibly unreliable [27], they were completed with prevalence-adjusted bias-adjusted kappa for dichotomous categorical data [28]. On the other hand, the sample was representative of shunted iNPH patients.

### Clinical implications

Imaging is important for the diagnosis of iNPH, and early detection of the disease facilitates prompt treatment. With corresponding reliability for CT and MRI, when it comes to reporting signs of iNPH, radiologists and neurologists can be confident in the assessment independent of modality. For detection of iNPH, CT is a suitable first-line investigation because of its wide availability and low cost, whereas MRI can confirm the diagnosis and rule out other causes of ventriculomegaly. The iNPH Radscale includes seven iNPH-associated features and can aid the structured reporting. Other scales and scoring systems have been suggested, i.e., the iNPH probability calculator for diagnosis and the DESH score for selection of shunt candidates [17, 29]. The iNPH probability calculator includes Evans’ index and callosal angle and has been proven to discriminate Alzheimer’s disease from iNPH. However, the DESH features are not included, with the risk of false negative results. DESH score is constructed for predicting shunt response, which is important to separate from diagnostic purposes.

Reporting a standardized measurement that is assessed in an arbitrary way could potentially mislead an investigation. If it is not performed correctly, such a measurement should not be reported at all. A standardized measurement procedure can be time-consuming and requires patience, but hopefully it produces reproducible data and eliminates reader-dependent/subjective reports.

## Conclusions

CT and MRI are equally good for assessing the radiological signs associated with iNPH. The agreement was substantial to almost perfect, except for periventricular white matter changes, as MRI has superior soft tissue contrast. As with any semiquantitative assessment, thorough alignment and harmonization between readers is essential. Therefore, the iNPH Radscale is applicable to both CT and MRI and may become an important tool for standardized evaluation in the workup in patients with suspected iNPH.

## Electronic supplementary material

ESM 1 (PDF 5732 kb)
