# Supplementary material for: Standardized image evaluation in patients with idiopathic normal pressure hydrocephalus: consistency and reproducibility
Source: Neuroradiology. 2019 Aug 10;61(12):1397–406. doi: 10.1007/s00234-019-02273-2 (PMC6848037; doi:10.1007/s00234-019-02273-2)
Supplement: Supplementary file 1 — (PDF 5732 kb) [file 234_2019_2273_MOESM1_ESM.pdf]

	0	1	2
**Evans' index**<br><br>0 = ≤ 0.25<br>1 = > 0.25 – 0.3<br>2 = > 0.3	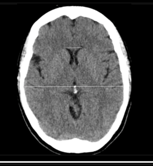	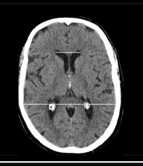	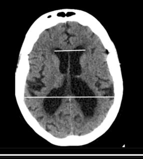
**Narrow Sulci**<br><br>0 = Normal<br>1 = Parafalcine<br>2 = Vertex	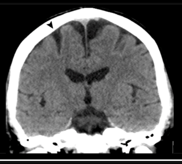	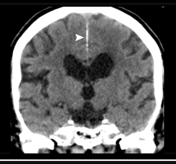	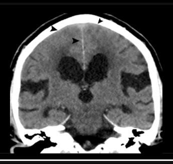
**Sylvian Fissures**<br><br>0 = Normal<br>1 = Enlarged	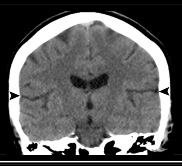	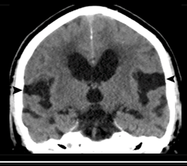	
**Focally enlarged sulci**<br><br>0 = Not present<br>1 = Present	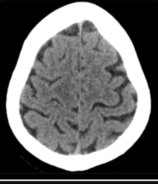	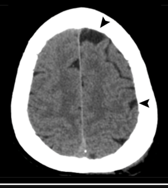	
**Temporal horns**<br><br>0 = < 4 mm<br>1 = 4 - < 6 mm<br>2 = ≥ 6 mm	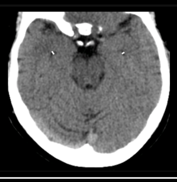	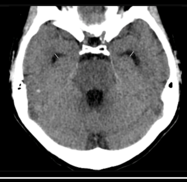	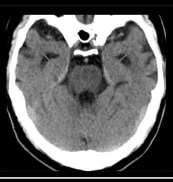
**Callosal angle**<br><br>0 = > 90°<br>1 = 90° - > 60°<br>2 = ≤60°	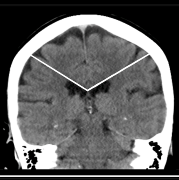	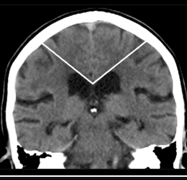	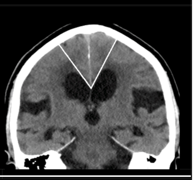
**Periventricular hypodensities**<br><br>0 = Not present<br>1 = Frontal horn caps<br>2 = Confluent areas	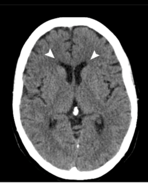	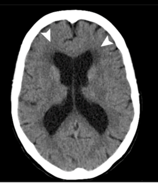	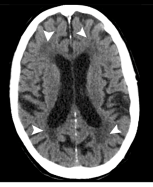
